# Immunosenescence and metabolic reprogramming in MASLD: an age-dependent immunometabolic vicious cycle and therapeutic opportunities

**DOI:** 10.3389/fcell.2025.1650677

**Published:** 2025-10-08

**Authors:** Yuxin Xu, Qiuxiang Li, Xuehua Jiao

**Affiliations:** 1 Department of Endocrinology, Suzhou Ninth People’s Hospital, Suzhou Ninth Hospital Affiliated to Soochow University, Suzhou, China; 2 The Second Clinical Medical College, Nanjing Medical University, Nanjing, Jiangsu, China

**Keywords:** metabolic dysfunction-associated steatotic liver disease (MASLD), immunosenescence, metabolic reprogramming, vicious cycle, mitochondrial dysfunction, combination therapy

## Abstract

Metabolic Dysfunction-Associated Steatotic Liver Disease (MASLD) poses a disproportionately severe burden on the aging population, with a heightened risk of progression to advanced fibrosis and cancer. While immunosenescence and metabolic reprogramming are recognized as key drivers, this review proposes an age-dependent immunometabolic vicious cycle as a critical integrative framework underlying MASLD progression. We hypothesize that at the core of this cycle lies mitochondrial dysfunction and reactive oxygen species (ROS) accumulation, which may initiate a self-amplifying loop: triggering NLRP3 inflammasome activation in Kupffer cells, promoting a context-dependent dysfunction of adaptive immunity. This includes driving CD8^+^ T cells toward exhaustion in advanced disease and disrupting regulatory T cell (Treg) function, which may range from loss of suppressive capacity to a pro-fibrotic phenotypic switch. Together, these alterations in T cell immunity create a permissive environment for unchecked inflammation and fibrosis. This cycle is further reinforced by gut-liver axis dysfunction. Critically, this framework reveals that overcoming the therapeutic bottleneck in age-associated MASLD necessitates a paradigm shift toward combination therapies that simultaneously target multiple nodes of the cycle.

## 1 Introduction

Metabolic Dysfunction-Associated Steatotic Liver Disease (MASLD) is a diverse liver disease occurring in the absence of other apparent etiologies and its pathogenesis is complex, involving the interaction of multiple factors (Buzzetti et al., 2016). It is characterized by hepatic lipid accumulation, lipotoxicity, insulin resistance, intestinal dysbiosis, and inflammation (Tilg et al., 2021). With the intensification of global population aging, MASLD has become prevalent worldwide, and its prevalence has shown a significant upward trend, especially among the elderly population, the likelihood of developing advanced liver conditions, including fibrosis, cirrhosis, and potentially hepatocellular carcinoma (HCC), is significantly elevated. This is particularly relevant given that MASLD has been robustly identified as an independent and important risk factor for HCC development, a concern that is further amplified in the context of aging (Zhu et al., 2025). At present, there are no precision drugs for MASLD. The primary approach to treatment involves altering one’s lifestyle, but it is challenging for patients to maintain this lifestyle in the long term.

Immunosenescence (the age-related functional decline of the immune system) and metabolic reprogramming (the adaptive rewiring of cellular metabolism) are key drivers of age-associated MASLD, fostering a state of chronic low-grade inflammation and metabolic stress (Miao et al., 2024). Senescent immune cells exhibit a reduced capacity to clear pathogens and debris while autonomously secreting pro-inflammatory factors.

MASLD does not affect all age groups equally; it disproportionately burdens the elderly population. It is not merely a higher prevalence among older adults but also an accelerated disease progression. Older patients exhibit a significantly greater risk of developing advanced fibrosis, cirrhosis, and HCC compared to their younger counterparts with the same condition. Moreover, MASLD is not confined to hepatic pathology; it is closely intertwined with other age-related health conditions (Younossi et al., 2025). This includes conditions such as sarcopenia (losing muscle mass and strength as you age), frailty (when your body handles stress worse, so small issues hit harder), and multimorbidity (having multiple chronic conditions like type 2 diabetes or heart disease). These factors collectively complicate the management of MASLD in elderly patients. Importantly, MASLD and these comorbidities share common pathophysiological drivers, such as insulin resistance and chronic low-grade inflammation. That creates a vicious cycle: MASLD worsens those other problems, and those problems speed up MASLD’s progression, all while hurting overall health. Therefore, to help older adults with MASLD, we can’t just focus on the liver—we have to look at the whole picture, including these age-related issues.

Studies have shown that immunosenescence and metabolic reprogramming are the primary drivers of age-associated MASLD (Zhang et al., 2021). The pathogenesis of MASLD has been viewed through several mechanistic lenses. While the ‘multiple-hit hypothesis’ provides a valuable framework, it may underrepresent the centrality of immune-metabolic crosstalk in aging. Similarly, the ‘gut-liver axis hypothesis’ highlights microbial contributions but often as a parallel pathway. Building on these foundations, we propose a novel synthesis: an age-dependent immunometabolic vicious cycle that integrates these elements into a self-propelling feedback loop.

**FIGURE 1:**
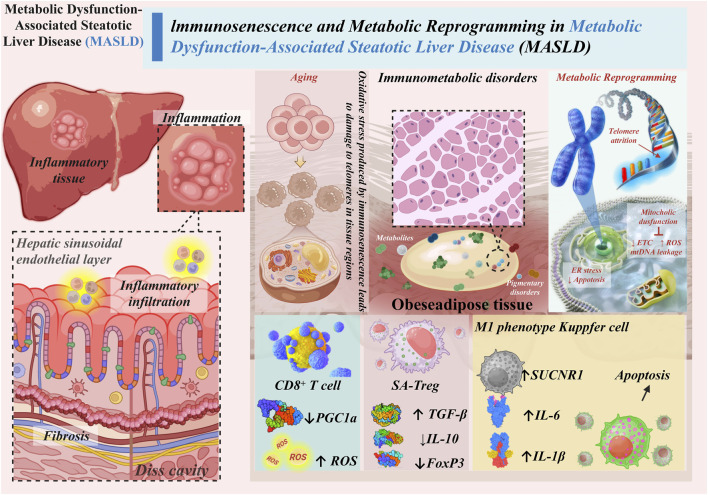
The age-dependent immunometabolic vicious cycle in MASLD.

This review moves beyond a descriptive account of these processes to propose and elucidate an age-dependent immunometabolic vicious cycle—a self-propelling feedback loop centered on mitochondrial dysfunction that amplifies hepatic injury and systemic inflammation. We will detail the evidence for this cycle and highlight how targeting its key nodes offers novel strategies for breaking the therapeutic bottleneck in MASLD (He et al., 2025). Figure 1 illustrates the vicious cycle of immunometabolic dysfunction that propagates MASLD progression in the aging liver.

Age-related triggers such as telomere attrition, lipotoxicity, and gut dysbiosis induce mitochondrial dysfunction in hepatocytes and immune cells. This leads to ROS accumulation, NLRP3 inflammasome activation, CD8^+^ T cell exhaustion, and Treg dysfunction, creating a self-reinforcing loop of inflammation and fibrosis. Solid arrows represent promoting effects; dashed arrows represent feedback reinforcement.

## 2 The initiation and core of the cycle

As outlined in the introduction, the age-dependent immunometabolic vicious cycle in MASLD is initiated through the convergence of multiple age-related pathological factors. Specifically telomere attrition in hepatic cells reduces regenerative capacity and promotes cellular senescence, while concurrent adipose tissue expansion leads to lipotoxicity through excessive free fatty acids (FFAs) release and adipokine dysregulation. Simultaneously, gut dysbiosis disrupts intestinal barrier function, enabling microbial translocation and endotoxemia (Tilg et al., 2022). These three fundamental aging processes collectively establish a state of persistent metabolic stress and chronic low-grade inflammation. This state, in turn, creates the essential pathological foundation that initiates hepatic mitochondrial dysfunction. This multifactorial trigger mechanism establishes the initial conditions necessary for cycle propagation, with each element reinforcing the others through interconnected feedback pathways (Kuang et al., 2023).

At the core of this self-amplifying cycle lies mitochondrial dysfunction, which serves as the central hub integrating and amplifying pathological signals. The combined impact of aging triggers disrupts mitochondrial structural integrity through lipid peroxidation and protein damage, while simultaneously suppressing mitochondrial biogenesis via PGC-1α downregulation. These alterations severely compromise electron transport chain (ETC) function, resulting in diminished ATP production, enhanced electron leakage, and consequent reactive oxygen species (ROS) overproduction. The resulting oxidative stress further damages mitochondrial components, creating a positive feedback loop that exacerbates mitochondrial impairment. Additionally, mitochondrial DNA leakage occurs due to membrane destabilization, releasing damage-associated molecular patterns (DAMPs) that activate pattern recognition receptors and initiate innate immune responses, thereby connecting metabolic dysfunction to inflammatory activation (Almalki and Almujri, 2024).

ROS generated from mitochondrial dysfunction act as critical signaling mediators. They propagate cellular damage across the hepatic microenvironment, thereby amplifying disease progression. Hepatocyte-derived ROS directly promotes immune cell senescence and activation, while senescent immune cells in turn release pro-inflammatory cytokines that further impair hepatocyte mitochondrial function (Liu et al., 2025). This reciprocal crosstalk establishes a self-reinforcing loop wherein metabolic dysfunction and immune activation mutually exacerbate each other, creating the fundamental feedback mechanism that drives MASLD progression. The persistent oxidative stress also induces endoplasmic reticulum stress, promotes lipid peroxidation, and activates cell death pathways, collectively contributing to hepatocellular injury and inflammation. This interconnected network of mitochondrial failure and oxidative damage forms the essential pathological engine that powers the continuous cycling and amplification of liver injury in age-related MASLD (Takubo et al., 2000).

## 3 The self-amplifying loop of the cycle

Building upon mitochondrial dysfunction and ROS accumulation, the vicious cycle progresses through dysregulation of both innate and adaptive immunity (Billingham et al., 2022). Mitochondrial damage (ROS/mtDNA leakage) activates the NLRP3 inflammasome in Kupffer cells, leading to a metabolically activated pro-inflammatory state and sustained secretion of cytokines such as IL-1β and IL-6. This inflammatory milieu not only promotes hepatocyte injury directly but also imposes profound stress on adaptive immune cells (Ajith et al., 2024).

CD8^+^ T cells show impaired mitochondrial function caused by PGC-1α inhibition. This leads to ROS overproduction and drives their transition into an exhausted phenotype with reduced cytotoxicity. In parallel, Tregs lose their immunosuppressive function, characterized by reduced IL-10 and increased TGF-β secretion, thereby acquiring a pro-fibrotic profile. This dual impairment—loss of immune surveillance from CD8^+^ T cells and loss of regulatory control from dysfunctional Tregs—creates a permissive environment for persistent inflammation, hepatic stellate cell activation, and collagen deposition (Tsuchida and Friedman, 2017; Wallace et al., 2022).

The subsequent fibrotic remodeling distorts liver architecture and exacerbates hypoxia and metabolic stress, which in turn amplify mitochondrial dysfunction, thereby closing the self-reinforcing loop. Beyond Kupffer cells and T cells, other immune subsets also contribute to this cycle. Age-related alterations in B cells promote pro-inflammatory cytokine secretion, while natural killer (NK) cell cytotoxicity declines with aging, impairing the clearance of damaged hepatocytes (Burra et al., 2023). Moreover, mucosal-associated invariant T (MAIT) cells, which are responsive to microbial metabolites, display altered function in both aging and metabolic disease, providing a potential mechanistic link between gut dysbiosis and hepatic immune dysfunction. Collectively, these immune perturbations perpetuate the immunometabolic vicious cycle that drives MASLD progression in the elderly (Toubal et al., 2019).

## 4 Systemic propagation of the cycle

The immunometabolic vicious cycle in MASLD is not confined to the liver. Through the gut–liver–immune axis, it propagates systemically and drives multi-organ dysfunction. Age-associated gut dysbiosis disrupts bile acid homeostasis by reducing bile salt–transforming bacteria and impairing FXR/TGR5 signaling (Pabst et al., 2023). These alterations compromise glucose and lipid metabolism, weaken intestinal barrier integrity, and increase permeability. As a result, pathogen-associated molecular patterns (PAMPs), particularly lipopolysaccharides (LPS), translocate across the gut barrier, forming immune complexes that reach the liver via portal circulation.

Within hepatic sinusoids, Kupffer cells recognize and phagocytose these microbial-derived complexes through FcαR and FcγR engagement, initiating robust pro-inflammatory responses. This gut-derived signaling establishes a feed-forward loop in which hepatic inflammation further damages intestinal barrier function, sustaining chronic immune activation and promoting fibrogenesis (Ma et al., 2020).

**FIGURE 2:**
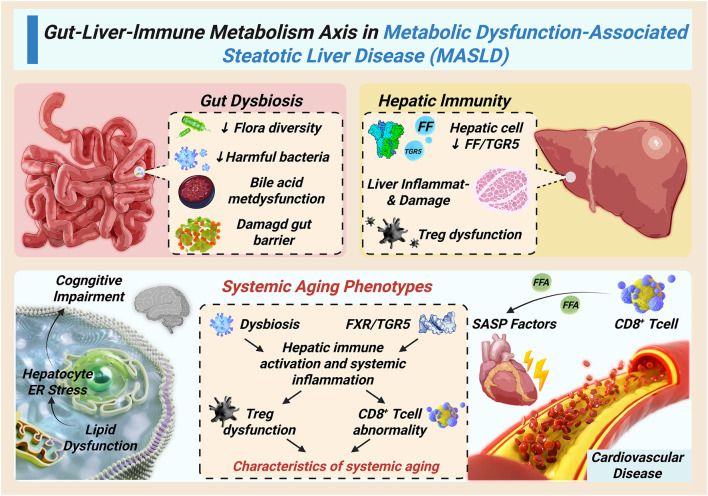
Systemic propagation of the immunometabolic vicious cycle through the gut-liver-immune axis.

At the same time, hepatic inflammation exerts systemic effects. Senescence-associated secretory phenotype (SASP) factors—including IL-6, TNF-α, and TGF-β—enter the circulation, driving endothelial dysfunction and atherosclerosis, impairing blood–brain barrier integrity and promoting neuroinflammation, as well as inducing insulin resistance, muscle atrophy, and bone loss in peripheral tissues (Libera et al., 2020; Kiourtis et al., 2024). These processes accelerate cardiovascular and neurodegenerative diseases while aggravating frailty and sarcopenia (Yao et al., 2021). Thus, the gut-liver-immune axis establishes a bidirectional and self-reinforcing pathway in which microbial dysbiosis, hepatic inflammation, and systemic aging phenotypes perpetuate each other, amplifying the progression of MASLD in elderly individuals. Figure 2 delineates the systemic propagation of the hepatic immunometabolic vicious cycle through gut-liver-immune crosstalk, elucidating how local liver inflammation escalates into multi-organ aging phenotypes.

Gut dysbiosis and impaired FXR/TGR5 signaling increase intestinal permeability, allowing microbial products to reach the liver and activate Kupffer cells. Hepatic inflammation, in turn, releases SASP factors that drive systemic aging phenotypes, including cardiovascular dysfunction, neuroinflammation, insulin resistance, and sarcopenia. This bidirectional gut–liver–immune crosstalk amplifies MASLD progression in the elderly.

## 5 Therapeutic approaches targeting the immune-metabolic cycle

The systemic and self-reinforcing nature of this immunometabolic vicious cycle presents a formidable therapeutic challenge. Consequently, effectively disrupting this cycle necessitates a paradigm shift from monotherapy to combinatorial strategies that simultaneously target multiple pathological nodes. This integrated approach should first address mitochondrial dysfunction at the cycle’s core. Compounds like Elamipretide, which stabilize mitochondrial membranes and reduce ROS production, hold promise (Tung et al., 2025). This can be complemented by NAD^+^ precursors such as Nicotinamide Mononucleotide (NMN) to enhance mitochondrial biogenesis and oxidative stress resistance, an approach supported by clinical trials showing improved insulin sensitivity in elderly MASLD patients (Allen et al., 2020).

Concurrent targeting of inflammatory amplification loops is essential. This can be achieved through dual-mechanism agents such as SGLT2 inhibitors, which offer metabolic benefits and exert anti-inflammatory effects by modulating exhausted CD8^+^ T cell populations (Liu et al., 2024). Chemokine receptor antagonists like cenicriviroc can be employed to block inflammatory monocyte recruitment and dampen innate immune activation. Simultaneously, peripheral drivers must be addressed through interventions targeting the gut-liver axis. FXR agonists can restore bile acid homeostasis, while microbiome-targeted therapies aim to enhance gut barrier function, thereby reducing microbial antigen translocation and subsequent hepatic inflammation.

However, the translational potential of the aforementioned strategies, predominantly validated in rodent models, requires critical appraisal. A significant “translational gap” exists, exemplified by the failure of several promising agents in human trials despite robust efficacy in mice. This disparity underscores the inherent limitations of preclinical models, which often inadequately recapitulate the chronicity, heterogeneity, and immunosenescent microenvironment of human aged MASLD. Therefore, while the combinatorial rationale is mechanistically sound, its success hinges on future validation in advanced humanized or aged animal models and, ultimately, well-stratified clinical trials (Midavaine et al., 2024).

The complexity of this immunometabolic network logically leads to these rationally designed combination therapies (Zhou et al., 2025). A strategic combination of mitochondrial protectants, immunomodulators, and gut-liver axis regulators offers the potential for synergistic effects that may effectively disrupt the self-amplifying nature of MASLD progression, providing superior clinical outcomes compared to conventional monotherapeutic approaches (Kritchevsky and Cummings, 2025).

## 6 Future challenges and research prospects

Translating the proposed immunometabolic vicious cycle from a theoretical framework into clinical practice presents several key challenges and opportunities. A primary and foundational hurdle is bridging the translational gap between preclinical models and human disease. This necessitates not only the validation of this model in humans across the aging spectrum but also a critical re-evaluation of the experimental systems we use (He et al., 2025). The frequent failure of therapies that are robust in mice demands the development of next-generation animal models that incorporate aging as a central factor, multi-system comorbidity, and humanized immune systems to faithfully mirror the human condition.

This validation requires longitudinal studies in elderly cohorts that track immune and metabolic parameters—such as mtDNA damage, NLRP3 activity, and T cell exhaustion markers—alongside MASLD progression, prioritizing human data derived from multi-omics approaches (single-cell RNA sequencing, spatial transcriptomics, metabolomics).

Furthermore, critical aging-specific questions remain unanswered: Does a critical threshold of mitochondrial dysfunction exist in immune cells beyond which this cycle becomes irreversible? Can we develop biomarker panels to dynamically monitor an individual’s position within this cycle and guide personalized therapy, thereby enabling better patient stratification for clinical trials?

Furthermore, the specific contributions of understudied immune populations - particularly B cells, senescent NK cells, and MAIT cells - within the aged MASLD microenvironment represent another critical knowledge gap that warrants thorough investigation (Soto-Heredero et al., 2025). Crucially, this cycle cannot be viewed in isolation. Future research must define its interplay with geriatric syndromes. It is essential to investigate how SASP factors from the liver exacerbate extrahepatic aging phenotypes like sarcopenia and frailty, and conversely, how pre-existing frailty may amplify the hepatic cycle through reduced metabolic reserve. The application of single-cell and spatial transcriptomics will be pivotal in addressing these questions, enabling the precise mapping of immune cell heterogeneity and intercellular communication within the aged MASLD liver microenvironment.

Beyond these challenges, emerging therapies offer novel pathways to disrupt this cycle. Senolytics target the inflammatory SASP by clearing senescent cells, directly mitigating a key cycle amplifier. Mitophagy inducers aim to restore mitochondrial quality control, fortifying the core against metabolic insult. Concurrently, late-stage clinical trials are advancing potent microbiota-targeted therapies, including engineered probiotics and phage-based approaches, to silence gut-liver axis activation. Integrating these strategies—targeting senescence, mitochondrial health, and dysbiosis—into combinatorial regimens represents the next frontier for breaking the vicious cycle in aged MASLD.

Ultimately, overcoming these challenges will pave the way for moving beyond histology-based staging toward an “immunometabolic staging” system, which is essential for crafting effective, multi-targeted interventions for the aging population.

## 7 Conclusion

In conclusion, by integrating current evidence from immunology and metabolism, we have constructed a conceptual model centered on an age-dependent immunometabolic vicious cycle. This framework is presented not as a definitive conclusion, but as a tool to rationalize clinical observations and guide future research for understanding the accelerated progression of MASLD in the elderly. Central to this model is the premise that mitochondrial dysfunction acts as a pathological hub, functionally linking immunosenescence with metabolic reprogramming and fibrosis. This cycle is perpetuated by intracellular and inter-organ crosstalk, most notably via the gut-liver axis, which systemically disseminates inflammation and contributes to multi-organ aging.

While the immunometabolic cycle framework provides an integrative explanation, it does not preclude alternative mechanisms. For instance, some evidence suggests that age-related lipotoxicity may be the primary driver, with immune dysfunction being a secondary consequence. Our model argues that these processes are inextricably linked and mutually reinforcing (Afonso et al., 2024). Furthermore, this cycle may not be universally applicable, highlighting the need for patient stratification based on dominant drivers. Future research should aim to reconcile these viewpoints.

The strength of this model lies in its explanatory power for clinical observations—such as the rapid fibrosis progression and high comorbidity burden in older patients—and its direct implication for therapeutic development. It compellingly argues that monotherapies are destined to fail as they target only a single node in a self-reinforcing network (De Carlo et al., 2025). Instead, breaking this cycle necessitates rational combination strategies that concurrently target mitochondrial integrity, immune inflammation, and gut microbiome ecology (Kim and Dixit, 2025).

However, several aging-specific knowledge gaps must be addressed to translate this framework into clinical practice. First, is there a critical threshold of mitochondrial function beyond which this cycle becomes irreversible? Second, how do pre-existing age-related conditions like sarcopenia and frailty interact with and amplify this hepatic cycle? Third, can we define biomarker panels to dynamically stage patients within this cycle and guide personalized therapy?

Future research employing single-cell multi-omics and spatial transcriptomics on aged human livers across the MASLD spectrum is crucial to validate the spatial and cellular interactions predicted by this mode. Ultimately, moving from a histology-based to an immunometabolic-based staging system represents the next frontier for developing effective, personalized interventions that can halt—rather than just slow—the progression of age-related MASLD.
