# Correction: Immunosenescence and metabolic reprogramming in MASLD: an age-dependent immunometabolic vicious cycle and therapeutic opportunities

**DOI:** 10.3389/fcell.2026.1849677

**Published:** 2026-05-12

**Authors:** Yuxin Xu, Qiuxiang Li, Xuehua Jiao

**Affiliations:** 1 Department of Endocrinology, Suzhou Ninth People’s Hospital, Suzhou Ninth Hospital Affiliated to Soochow University, Suzhou, China; 2 The Second Clinical Medical College, Nanjing Medical University, Nanjing, Jiangsu, China

**Keywords:** metabolic dysfunction-associated steatotic liver disease (MASLD), immunosenescence, metabolic reprogramming, vicious cycle, mitochondrial dysfunction, combination therapy

There was a mistake in Figure 1 as published.

**FIGURE 1:**
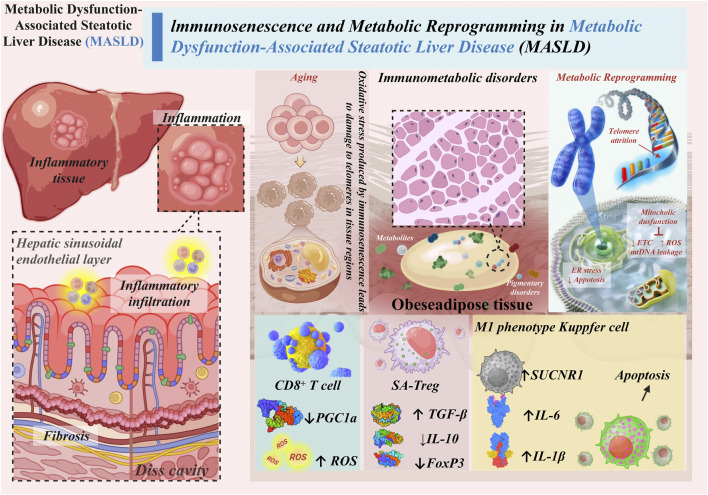
The age-dependent immunometabolic vicious cycle in MASLD.

The figure was created by us as an original schematic illustration. In preparing it, we referred to existing educational and reference materials for the general visual style of cell differentiation and morphology, and then produced our own schematic elements. However, although the figure was intended as an original cartoon-style illustration rather than a direct reproduction of any published figure, we recognize that the visual similarity of some parts is too high and may reasonably give rise to copyright or originality concerns. This was an oversight on our part, and we sincerely acknowledge the mistake.

The corrected Figure 1 appears below.

The original article has been updated.

